# Fluorescence Method–Based Nonlinear Hybridization Chain Reaction for Highly Sensitive and Specific Detection of HER2

**DOI:** 10.1155/ianc/3877339

**Published:** 2026-07-08

**Authors:** Feifei Liu, Jing Li, Wenna Jiang, Jing Pang, Shuo An, Yue Shi, Li Ren

**Affiliations:** ^1^ Department of Laboratory, Tianjin Medical University Cancer Institute & Hospital, National Clinical Research Center for Cancer, Tianjin′s Clinical Research Center for Cancer, Tianjin′s Clinical Research Center for Cancer, Key Laboratory of Cancer Prevention and Therapy, Tianjin, China, tijmu.edu.cn

**Keywords:** fluorescence sensor, gastric cancer, Human epidermal growth factor receptor 2, nonlinear hybrid chain reaction

## Abstract

Human epidermal growth factor receptor 2 (HER2) is a critical biomarker for targeted therapy of gastric cancer. Tissue‐based HER2 testing via immunohistochemistry (IHC) and fluorescence in situ hybridization (FISH) remains the clinical gold standard, while serum HER2 represents a potential complementary circulating biomarker for disease monitoring. However, conventional serum HER2 assays often suffer from limited sensitivity. Herein, we develop a highly sensitive fluorescence assay for serum HER2 detection using nonlinear hybridization chain reaction (NHCR) coupled with a HER2‐specific aptamer (HB5). Unlike linear hybridization chain reaction (HCR), NHCR enables branching‐mediated exponential amplification, in which each trigger initiates multiple independent HCR cascades, leading to nonlinear signal growth. The method exhibited a good linear relationship (*R* ^2^ = 0.993) in the HER2 concentration range of 0.5–90 ng·mL^−1^, with a detection limit as low as 38 pg·mL^−1^. The sensor demonstrated excellent specificity for HER2, good reproducibility, and reliable stability. Clinical serum sample detection results indicated that the method was highly correlated with the clinically commonly used chemiluminescence immunoassay (CLIA), with spiked recoveries ranging from 95.4% to 109.6%. The NHCR‐based fluorescent detection strategy established in this study provides a new tool for accurate and noninvasive detection of serum HER2, featuring simple operation and controllable cost.

## 1. Introduction

Gastric cancer is a prevalent malignant tumor globally, and patients with advanced disease often face a poor prognosis [1, 2]. Human epidermal growth factor receptor 2 (HER2) represents a critical therapeutic target in gastric cancer, and its overexpression guides anti‐HER2 therapy [3–5]. Tissue HER2 testing via immunohistochemistry (IHC)/ fluorescence in situ hybridization (FISH) using endoscopic biopsy specimens remains the clinical standard [6]. However, repeated endoscopy and biopsy may be limited in some patients, and serial monitoring is challenging using tissue alone [7, 8]. Serum HER2 (extracellular domain [ECD]) has emerged as a circulating biomarker candidate; while not currently recommended as a replacement for tissue HER2 testing in clinical guidelines, it may serve as a complementary tool for disease monitoring [9]. Furthermore, research has indicated that serum exosomes HER2 levels can be used as an effective biomarker to evaluate tissue HER2 status in patients with advanced gastric cancer and to screen patients with potential benefit from trastuzumab therapy [10–13]. Established serum HER2 detection methods include chemiluminescence immunoassay (CLIA) and enzyme‐linked immunosorbent assay (ELISA), whose performance depends heavily on antibody quality and can be limited by sensitivity and specificity [14].

Wang et al. [15] developed an electrochemical biosensor using an antifouling polypeptide hydrogel to detect HER2 in human serum with a detection limit of 45 pg·mL^−1^. Similarly, Li et al. [16] designed a polycytosine DNA immunosensor for electrochemical detection of serum HER2 with a linear range of 1 pg·mL^−1^ to 1 ng·mL^−1^. While these sensors have the advantages of being fast, robust, and capable of detecting multiple analytes, the complex process of synthesizing and modifying metal sensors often requires multiple reagents and time‐consuming substrates, making it costly. Compared to traditional antibodies, aptamers possess prominent merits, including easy synthesis, low immunogenicity, and stable binding performance, which have been widely applied in tumor biomarker targeted recognition [17–19].

On this basis, aiming to break through the bottlenecks of existing detection technologies, we innovatively introduce a nonlinear hybridization chain reaction (NHCR) with efficient cascade signal amplification capability [20–26]. In a typical linear hybridization chain reaction (HCR), two metastable hairpin probes are cross‐opened by an initiator and polymerize into a long linear nicked duplex, following linear growth kinetics [27–30]. By contrast, NHCR adopts rationally designed multibranched or dendritic probes (hairpins or double‐stranded substrates with toehold and loop domains) [31, 32]. Upon recognition of the target, the initiator triggers a cascade of toehold‐mediated strand displacement (TMSD) reactions, opening multiple probes simultaneously and driving the self‐assembly of branched, hyperbranched, or dendritic DNA nanostructures [33–39].

Our team [40] previously combined NHCR technology with aptamers to greatly improve the fluorescence amplification sensitivity of visfatin detection in serum. The incorporation of magnetic beads allows for quick separation of nonspecific fluorescence signals, resulting in a low detection limit of 0.028 ng·mL^−1^.

In this study, based on the previously reported HER2 aptamer sequence [41], we rationally optimized the number of toehold bases and carefully refined four hairpin structures through iterative design and screening. This deliberate design effectively prevented probe leakage and spontaneous self‐assembly in the absence of the target, thereby reducing background interference. Meanwhile, the optimized hairpin configuration guaranteed high sensing sensitivity and efficient cascade activation when specifically recognizing the HER2 target. By constructing a standard curve of fluorescence spectrum and the logarithm of target protein concentration, we were able to accurately monitor HER2 levels. In the absence of the target, the stable coexistence of NLH1, NLH2, NLH3, NLH4, and the trigger aptamer double chain prevented spontaneous assembly, and the fluorescence signal could be detected scarcely by the spectrophotometer. The application of aptamers to the NHCR system could ensure that the NHCR generated during the detection process was limited to the target molecule, thus increasing the sensitivity and reducing the false‐positive rate. The entire reaction proceeded spontaneously at a constant temperature without requiring enzymes or thermal cycling, which simplified experimental operation and provided a reliable theoretical and technical basis for the application of the NHCR strategy in HER2‐related clinical molecular detection.

## 2. Materials and Methods

### 2.1. Reagents

All reagents were of high purity, and the solutions were prepared in ultrapure water. Nucleic acid probes were synthesized by Sangon Biotechnology Co., Ltd. (Shanghai, China), and the designed base sequences are shown in Table S1. The ECD of the HER2 protein was purchased from Abcam, USA. The DNA Ladder Marker was provided by Union Biotech Co., Ltd. Sodium chloride (purity standard material 99.96% ± 0.02%), dipotassium hydrogen phosphate (chromatographic grade, ≥ 99.0% (T)), and magnesium chloride were purchased from Aladdin Reagent Co., Ltd. (Shanghai, China). The human serum albumin and insulin were purchased from Sigma‐Aldrich (Shanghai, China). Human HER2 detection kit was purchased from Shenzhen New Industry Biomedical Engineering Co., Ltd. All procedures involving human samples in this study were conducted in accordance with the principles of the Declaration of Helsinki and were reviewed and approved by the Ethics Committee of Tianjin Medical University Cancer Hospital (EK20240370).

### 2.2. Instruments

Fluorescence physical properties were characterized by an RF6000 fluorescence spectrophotometer (Shimadzu, Tokyo, Japan). The morphology of the final product was characterized by atomic force microscopy (Brucker, Germany), polyacrylamide gel electrophoresis (PAGE) was performed on a JY1000C electrophoresis instrument (Beijing Junyi‐Oriental Electrophoresis Instrument Co., Ltd. https://www.bjjuyi.com/), and imaging was performed using the Bio‐Rad ChemDocXRS imaging system (Bio‐Rad Laboratories, USA).

### 2.3. Preparation of Probes

In reference to a recent study on the HER2 aptamer sequence [41], the probe sequence (Table S1) for the NHCR system was designed and optimized. The fluorescent group (FAM) and quenching group (BHQ1) were labeled at both ends of the hairpin chain. The purified base sequence was dissolved in 1 ∗ TE buffer (10 mM Tris‐HCl and 0.5 mM EDTA, pH 8.0). A mixture of trigger DNA (10 μM) and HER2 aptamer (10 μM) was heated to 95°C for 2 min and then gradually cooled to room temperature over 30 min, resulting in partial hybridization of the trigger DNA and HER2 aptamers. This solution was referred to as “Solution 1.” The corresponding NLH1‐NLH4 chains and H1and H2 chains (10 μM) were heated to 95°C for 2 min [42] and then cooled to room temperature to obtain “Solutions 2–7.” These solutions were stored at 4°C for further experiments. Meanwhile, a PBS‐based working buffer containing 5 mM MgCl_2_ and 2.5 mM K_2_HPO_4_ was freshly prepared and used to configure the subsequent 50 μL total reaction system.

### 2.4. Synthesis of NHCR Reaction System

First, the HER2 protein solution was mixed with Solution 1 (10 μM) at room temperature for 30 min, which resulted in specific binding to the aptamer sequence and exposed the trigger chain sequence. This trigger chain opened the structure of Hairpin 1 and exposed a new toehold for further reaction with the added Hairpin 2. As the hairpin structure continued to open, the distance between the FAM and BHQ1 at both ends of the hairpin chain increased, leading to the production of a fluorescence signal. The opened Hairpin 2 then initiated a hybrid chain reaction with the added Hairpin 3 and Hairpin 4, generating more fluorescence signals and exposing new toeholds. This process resulted in a cross‐shaped branching structure with a toehold at each branch, and each toehold acted as a trigger switch to initiate a new round of hybrid chain reaction. Therefore, in the NHCR reaction system, the fluorescence signal increased exponentially.

The reaction program was set on a PCR instrument as follows: 95°C for 2 min, followed by cooling to 38°C at a rate of 1°C/s, and then incubation at 38°C for 1 h to form the final products. A parallel control experiment was performed under the same reaction conditions without the presence of the HER2 protein to eliminate nonspecific reactions.

### 2.5. PAGE

To confirm the maneuverability of the self‐assembly system, the products of the NHCR process were studied using 8% natural PAGE. The NHCR system without a target protein was prepared (0 ng·mL^−1^ HER2 + aptamer + trigger + Hairpin 1+ Hairpin 2 + Hairpin 3 + Hairpin 4), as well as the NHCR system with different concentrations of detection target (10/25 ng·mL^−1^ HER2 + aptamer + trigger + Hairpin 1 + Hairpin 2 + Hairpin 3 + Hairpin 4). The hairpin chains and end‐product were mixed with 6 × loading buffer in a 5:1 volume ratio, respectively, and stored at 4°C for subsequent electrophoresis analysis. The samples were added to Holes 1–8 in the following order: Lane 1: 20 bp DNA Ladder Marker, Lane 2: NLH1, Lane 3: NLH2, Lane 4: NLH3, Lane 5: NLH4, Lane 6: end‐product without HER2 target, Lane 7: end‐product with 10 ng·mL^−1^ HER2 target, and Lane 8: end‐product with 25 ng·mL^−1^ HER2 target. After the NHCR, the nucleotides formed a large cross‐like structure with a high molecular weight (> 500 bp).

### 2.6. Fluorescence Characterization

According to the basic principle of NHCR reaction, cross‐like structures with numerous fluorescence signals were produced when NHCR occurred. In contrast, when NHCR did not occur, the hairpin chains maintained their original structures and were in a state of fluorescence quenching. Therefore, the feasibility and the amplification efficiency of the NHCR strategy could be further verified by monitoring the change in fluorescence intensity. The fluorescence intensity of the solution was measured using a fluorescence spectrophotometer with an excitation wavelength of 550 nm and an emission wavelength range of 560–700 nm. Both the excitation and emission slits were set to 3 nm.

### 2.7. HER2 Detection

After different amounts of HER2 (10 μL, 0.5, 1, 1.5, 5, 7.5, 20, 25, 45, and 90 ng·mL^−1^) were mixed with 10 μL of Solution 1 (10 μM) at room temperature for 30 min, 10 μL of Solutions 2–5, 10 μL of 5 mM MgCl_2_, and 30 μL of 2.5 mM K_2_HPO_4_ were added to the above mixture and incubated at 38°C for 1 h. During this process, trigger DNA was released from the HER2 aptamer duplex and triggered a series of self‐assembly behaviors of hairpin structures to form cross‐shaped DNA macromolecules with four directions, and then the fluorescence intensity of the solution was detected.

### 2.8. Comparison of CLIA and Fluorescence Detection Methods Using Gastric Cancer Patient Specimens

Taking the measured NHCR system fluorescence signals as the *Y* value, the concentration of HER2 in the measured blood sample was obtained by substituting *X* into the linear regression equation. The serum concentration of the fluorescence method was compared with the CLIA to evaluate the practicability and reliability of the proposed analysis strategy.

## 3. Results

### 3.1. Characterization of NHCR System End‐Product

**FIGURE 1:**
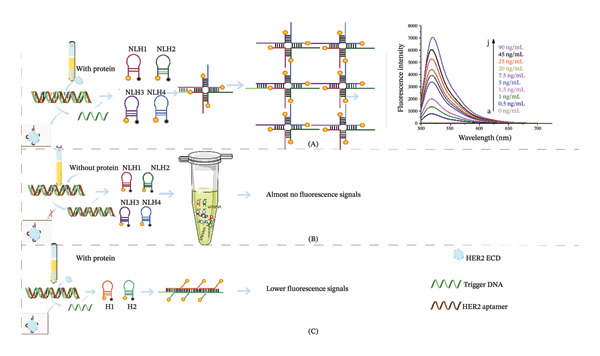
Schematic description of the detection process of the NHCR‐based HER2.

**FIGURE 2:**
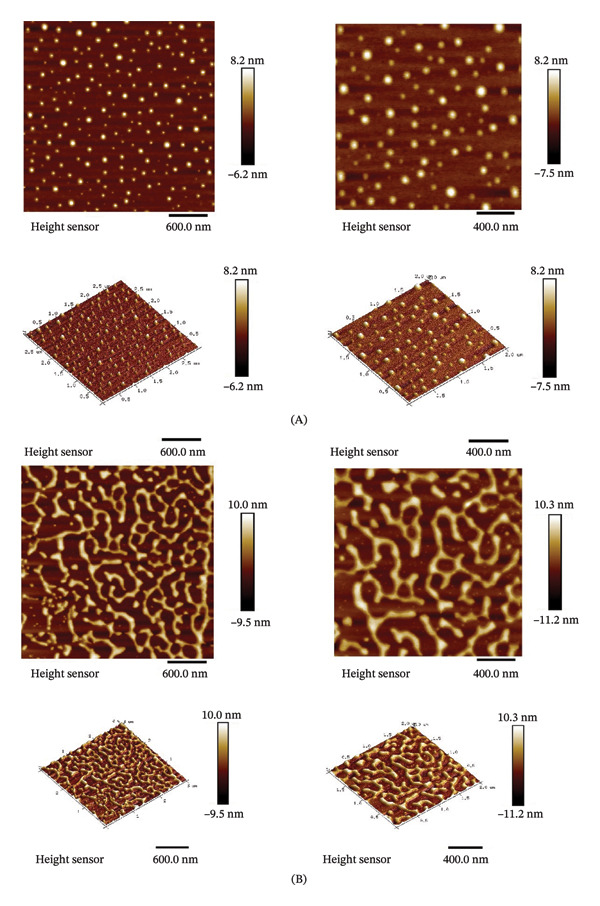
Image of the end‐product by atomic mechanics microscopy. (A) Atomic mechanics microscopy of products formed in the absence of HER2. (B) Atomic mechanics microscopy of products formed in the presence of HER2.

The reaction mechanism of NHCR is shown in Figure 1. The mixed solution formed under 0 ng·mL^−1^ HER2 and 50 ng·mL^−1^ HER2 was characterized using an atomic force microscope. Without the HER2 protein, the image revealed scattered structures, which were identified as unassembled hairpin structures and aptamer trigger chains (Figure 2A). In contrast, the presence of obvious branched structures (Figure 2B) confirmed the successful self‐assembly of the hairpin chains into the expected DNA macromolecules. The results demonstrated that after centrifugation at 11,000 rpm for 1 min, white flocculent precipitates were observed at the bottom of the right EP tube, while the left solution remained clear, which indicated that DNA cross‐like structures could be formed in the presence of detection targets (Figure S1).

### 3.2. Feasibility Verification of the NHCR System

**FIGURE 3:**
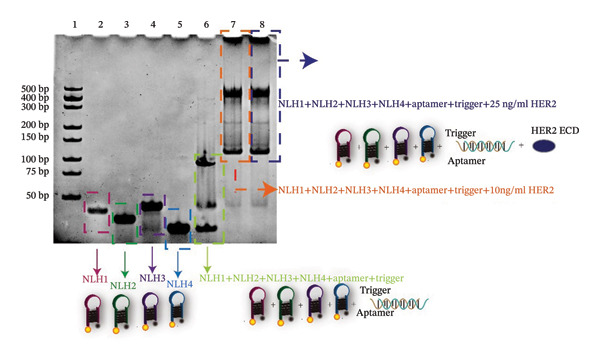
Natural polyacrylamide gel electrophoresis results of a nonlinear hybrid chain reaction. M is a DNA marker (20–500 bp).

The feasibility of the NHCR reaction system was verified through 8% native PAGE for 50 min at 120 V. The gel was then immersed in gold view dye solution for 15 min before being photographed using a Bio‐Rad digital imaging system. The PAGE results confirmed that the presence of different concentrations of HER2 successfully triggered the self‐assembly of NLH1, NLH2, NLH3, and NLH4, resulting in the formation of new bands with slower mobility and higher molecular weights (Lane 7 and Lane 8), confirming the smooth progression of the reaction system as expected (Figure 3). In the absence of HER2 (Lane 6), NLH1, NLH2, NLH3, NLH4, and aptamer double strands remained stable due to limitations in dynamics, demonstrating the excellent design of this reaction system.

### 3.3. Optimization of Experimental Parameters

**FIGURE 4:**
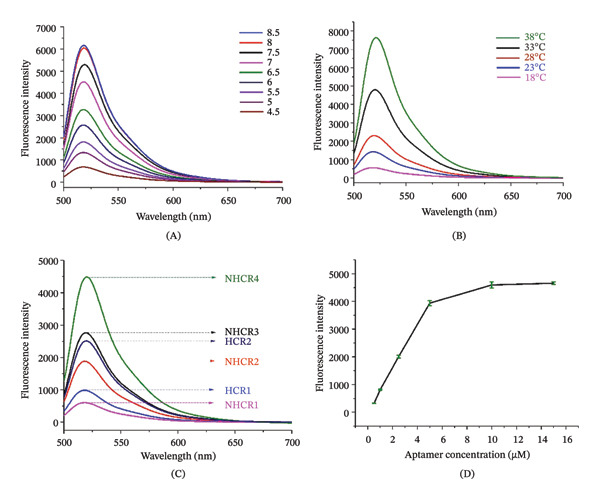
Optimization of experimental conditions for the NHCR system.(A) Fluorescence spectra recorded from the reaction system after incubating the aptamer with 10 μM HER2 at 38°C in a buffer of pH 4.5–8.5. (B) Fluorescence spectra recorded from the reaction system at 18°C–38°C temperatures of pH 8 for 1 h. (C) Characterization of the amplification efficiency with normal HCR and NHCR. (D) Fluorescence spectra recorded from the reaction system after incubating the solution with the aptamer of different concentrations at 38°C. Error bars indicate the mean ± standard deviation, *n* = 3.

To achieve optimal reaction performance, several key experimental conditions were carefully optimized. The optimal pH for the reaction system was determined to be 8. As the pH increased, the fluorescence intensity of the system also increased. However, once the pH exceeded 8, the change in fluorescence intensity became less significant, which suggested that the overly alkaline environment was not conducive to the occurrence of chain displacement reactions (Figure 4A). In addition, the reaction temperature was found to greatly impact the NHCR process. Temperatures that were too low hindered the self‐assembly reaction, while excessively high temperatures could denature the target protein and affect the reaction efficiency. The results indicated that the optimal temperature for the reaction was 38°C (Figure 4B). Furthermore, the amplification efficiency of NHCR was compared to that of linear HCR. There was an enhanced fluorescent response, which was obtained for the two indicators compared to one labeled NHCR. The fluorescent signal was further improved when three or four indicators were employed due to the high amplification efficiency of NHCR (Figure 4C). The one indicator– and two indicator–labeled HCR generated a lower fluorescent response, which demonstrated that whole indicators were extremely crucial for the sensitivity in the detection of NHCR. Under the above optimal conditions, we further explored the optimal aptamer concentration required for the reaction system to ensure the effective binding of aptamer and HER2 (Figure 4D).

### 3.4. Detection of HER2

**FIGURE 5:**
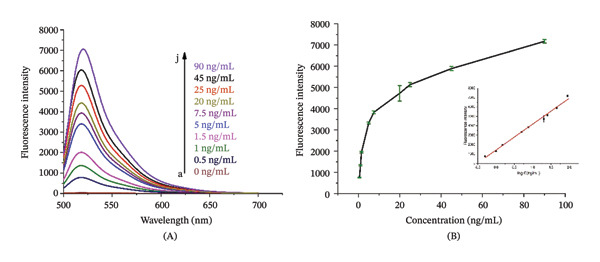
Linear relationship between fluorescence signal intensity and logarithm of HER2 concentration. (A) Fluorescent responses via NHCR for different concentrations of HER2 ranged from 0 to 90 ng·mL^−1^. (B) Calibration curve for the relationship between the fluorescent spectra and the logarithm of the concentration of HER2. Error bars indicate the mean ± standard deviation, *n* = 3.

The exponential amplification mechanism of NHCR was reflected in its reaction kinetics, where a single trigger chain could initiate multiple branching reactions, thereby conferring the fluorescence signal with exponential growth potential during the reaction. The detection performance of the fluorescence sensor for HER2 was evaluated under the best experimental parameters. The fluorescence signal increased with increasing HER2 concentration due to the high specificity of HER2 binding to its aptamer sequence (Figure 5A). In the range of 0.5–90 ng·mL^−1^ HER2 concentration, there was a strong linear relationship between the fluorescence signal and the logarithm of HER2 concentration. The linear regression equation is expressed as *Y* = 2754.4*X* + 1417.8 (*R* ^2^ = 0.993) (Figure 5B), where *X* = Log C. The detection limit of the biosensor was estimated to be 38 pg·mL^−1^ based on the 3*σ* rule. This was a good sensitivity compared to other reported HER2 detection methods (Table 1), indicating the excellent signal cascade amplification function of NHCR under suitable conditions, resulting in a sensitive fluorescence signal.

**TABLE 1:** Comparison between existing HER2 determination methods developed in this work.

Method	Linear range	Detection limit	Advantages	Limitations	Reference
Impedimetric immunosensor	10–100 ng·mL^−1^	7.2 ng·mL^−1^	Simple fabrication	Low sensitivity, narrow range	[43]
Amperometric magnetoimmunosensor	0.1–32 ng·mL^−1^	26 pg·mL^−1^	High sensitivity	Complex magnetic separation	[44]
Electrochemical biosensors	0.1 ng·mL^−1^–1 μg·mL^−1^	45 pg·mL^−1^	Wide range	Electrode modification required	[15]
Electrochemical aptasensor	10–60 ng·mL^−1^	3.0 ng·mL^−1^	Aptamer‐based	Low sensitivity	[45]
Fluorescence assay	0.5–90 ng·mL^−1^	38 pg·mL^−1^	Ultrasensitive, enzyme‐free, simple operation	Preliminary clinical validation	This work

### 3.5. Selectivity, Reproducibility, and Stability of HER2 Fluorescence Sensor

**FIGURE 6:**
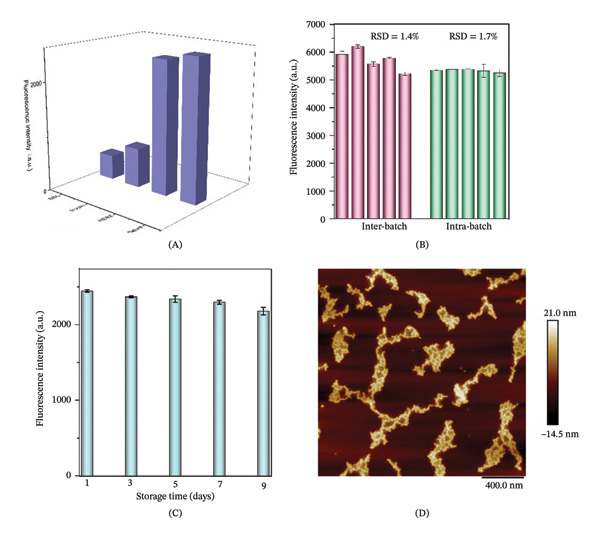
Verification of the specificity, repeatability, and stability of the reaction system. (A) Results obtained in the selectivity studies using the proposed strategies by incubation in the following solutions under the same conditions: BSA, insulin, (100 ng·mL^−1^ of each interfering substance), 1 ng·mL^−1^ HER2, and a mixture of these (*p* < 0.01). (B) Reproducibility of the developed assay with 40 ng·mL^−1^ HER2. (C) Storage stability of as‐prepared NHCR system with 50 ng·mL^−1^ HER2. (D) Morphological stability evaluated by AFM imaging of NHCR products. Error bars indicate the mean ± standard deviation, *n* = 3.

To verify the selectivity of the fluorescence analysis technique to the target protein, the fluorescence intensity of HER2, insulin, and bovine serum albumin (BSA) and the mixture of interferer and target protein were measured. Even at a concentration 100 times higher than HER2, these proteins only produced extremely weak signals compared to the target HER2, indicating the excellent specificity of the biosensor (Figure 6A). The reproducibility was then checked under the optimum reaction conditions by measuring the relative standard deviation (RSD) of interassay and intraassay signals. The RSDs were 1.4% and 1.7%, respectively, demonstrating acceptable repeatability (Figure 6B). There was no significant difference in fluorescence intensity within 9 days of storage, suggesting the trustworthy stability of the proposed detection method (Figure 6C). The products were subjected to morphological characterization using AFM following 1 week of storage. The nanostructure was observed to be well‐preserved, with clearly discernible branched structures (Figure 6D).

### 3.6. Clinical Application of Serum HER2 Detection

**FIGURE 7:**
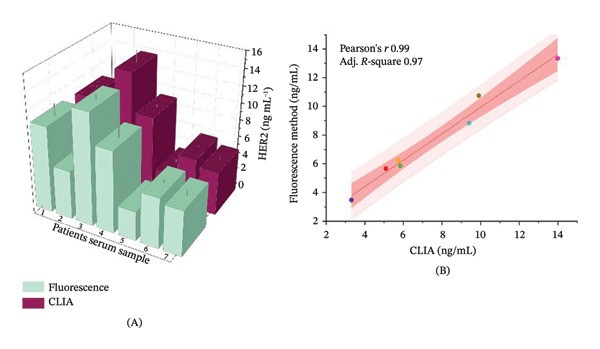
Comparison of reliability of two detection methods. (A) Detection of serum HER2 levels in patients with gastric cancer by chemiluminescence and newly developed fluorescence methods. (B) Scatter plot with linear regression analysis showing a strong correlation between the two methods. Error bars indicate the mean ± standard deviation, *n* = 3.

As a preliminary proof‐of‐concept study, seven gastric cancer serum samples were tested using fluorescence sensors and CLIA (Figure 7A). A strong correlation was observed in the linear regression analysis, which demonstrated good consistency between the two analytical approaches (Figure 7B). Moreover, serum samples collected from clinical settings were diluted, and various concentrations of HER2 (0.5, 1.5, and 6.5 ng·mL^−1^) were added. The calculated recovery rate ranged from 95.4% to 109.6% (Table 2), with an RSD of 0.42%–4.62%, which demonstrated that the fluorescent biosensor was suitable for detecting HER2 in serum.

**TABLE 2:** HER2 was detected by fluorescence assay in patients with gastric cancer.

Samples	Added	Found	Recovery (%)	RSD (%)
1	0.5 ng·mL^−1^	0.54 ng·mL^−1^	1.09	0.42
	0.5 ng·mL^−1^	0.55 ng·mL^−1^	1.10	
	0.5 ng·mL^−1^	0.55 ng·mL^−1^	1.10	

2	1.5 ng·mL^−1^	1.48 ng·mL^−1^	0.99	1.76
	1.5 ng·mL^−1^	1.43 ng·mL^−1^	0.95	
	1.5 ng·mL^−1^	1.45 ng·mL^−1^	0.97	

3	6.5 ng·mL^−1^	6.83 ng·mL^−1^	1.05	4.62
	6.5 ng·mL^−1^	6.29 ng·mL^−1^	0.97	
	6.5 ng·mL^−1^	6.80 ng·mL^−1^	1.05	

## 4. Conclusions and Discussion

The proposed method demonstrated a good linear response within the range of 0.5–90 ng·mL^−1^. The core innovation was integration of aptamer recognition with branching‐mediated exponential amplification of NHCR, which overcame the limited sensitivity of conventional serum HER2 assays [46].

Compared to state‐of‐the‐art methods (Table 1), our assay achieved a low LOD of 38 pg·mL^−1^, comparable to electrochemical sensors but with simpler operation and no electrode modification. However, the assay had limitations. First, it was a preliminary proof‐of‐concept study with a small clinical cohort (*n* = 7); larger cohorts including healthy controls and patients with different disease stages were needed to validate clinical utility. Second, serum matrix interference may affect performance in highly heterogeneous samples; further optimization of buffer conditions was required. Third, the available clinical records of these 7 gastric cancer serum samples lacked definitive HER2 amplification profiling data, and retrospective detection could not be performed due to the limitation of residual sample volume and clinical specimen conditions. We have acknowledged this as a limitation of the current study and suggested further validation with well‐characterized HER2‐stratified gastric cancer serum samples in future research. Circulating tumor DNA sequencing may eventually provide a more comprehensive noninvasive alternative, while our assay is intended as a sensitive tool for serum HER2 measurement and disease monitoring.

## Author Contributions

Feifei Liu: conceptualization and investigation. Jing Li: validation. Wenna Jiang: writing–original draft. Jing Pang: software. Shuo An: data curation. Yue Shi: methodology. Li Ren: funding acquisition and supervision.

## Funding

This work was supported in part by the Tianjin Health Science and Technology Project (TJWJ2022ZD003), the Tianjin Medical University Cancer Hospital Pharmacy, Laboratory, and Imaging Special Fund (Y2310), and the Tianjin Key Medical Discipline Construction Project (TJYXZDXK‐3‐003A).

## Disclosure

A preprint has previously been published in Research Square (DOI/archive link: https://doi.org/10.21203/rs.3.rs-7202602/v1).

## Ethics Statement

All procedures were performed in compliance with relevant laws and institutional guidelines and have been approved by Cancer Biobank of Tianjin Medical University Cancer Institute & Hospital (EK20240370), and the privacy rights of human subjects have been observed and that informed consent was obtained for experimentation with human subjects.

## Consent

Please see the Ethics Statement.

## Conflicts of Interest

The authors declare no conflicts of interest.

## Supporting Information

Additional supporting information can be found online in the Supporting Information section.

## Supporting information


**Supporting Information** All supporting data are available in the supporting information of this article. Supporting file 1: Figure S1. The products adding HER2 were centrifuged. Supporting file 2: Table S1. Oligonucleotide sequence used in this study.

## Data Availability

Data will be made available on request.
