# Supplementary material for: Fluorescence Method–Based Nonlinear Hybridization Chain Reaction for Highly Sensitive and Specific Detection of HER2
Source: Int J Anal Chem. 2026 Jul 8;2026:3877339. doi: 10.1155/ianc/3877339 (PMC13343312; doi:10.1155/ianc/3877339)
Supplement: Supplementary file 1 — Supporting Information All supporting data are available in the supporting information of this article. Supporting file 1: Figure S1. The products adding HER2 were centrifuged. Supporting file 2: Table S1. Oligonucleotide sequence used in this study. [file IANC-2026-3877339-s001.docx]

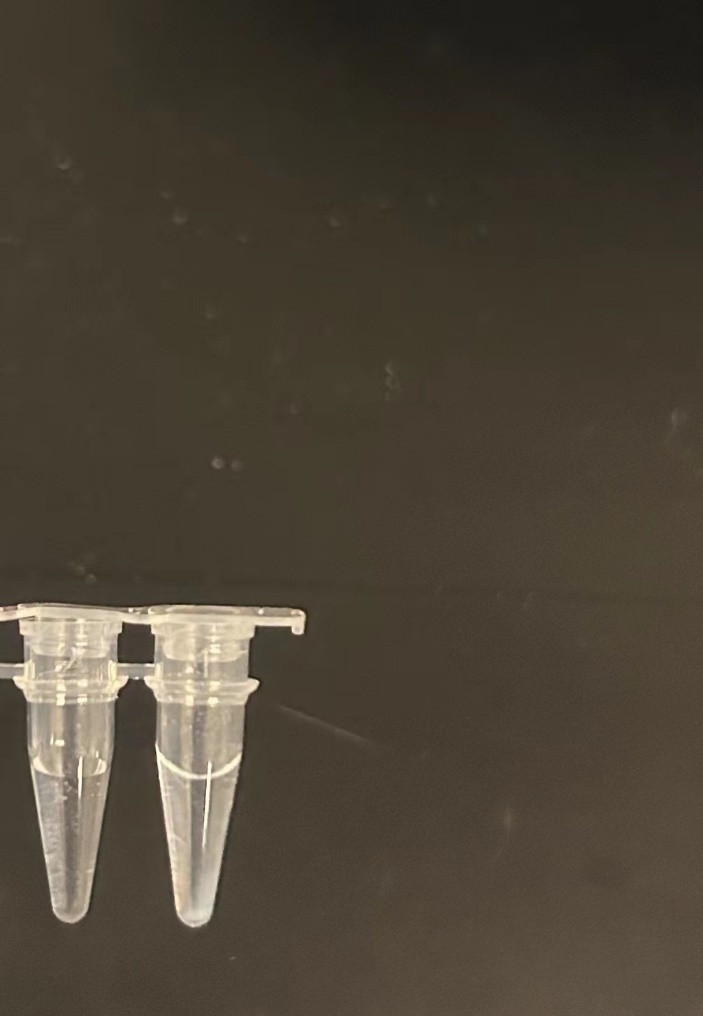


Fig. S1 The products without (left) or with (right) adding HER2were centrifuged under 11000 rpm for 1 min.

Table S1 Oligonucleotide sequence used in this study

| Oligonucleotide chain | 5’-3’Sequence |
| --- | --- |
| HER2 Aptamer | AA CCG CCC AAA TCC CTA AGA GTC TGC ACT TGT CAT TTT GTA TAT GTA TTT GGT TTT TGG CTC TCA CAG ACA CAC TAC ACA CGC ACA |
| Trigger DNA | TGT GCG TGT GTA GTG TGT CTG TGA GAG CCA AAA ACC AAA TAC ATA TAC AAA ATG ACA AGT GCA GAC TCT TAG GGA TTT GGG CGG TT AGG AAG TTT |
| NLH1 | TTT （FAM)CTT CCT AAC CGC TCT TCC CGA GGC GTA CCC CCC CGA GCT ACG AAG-BHQ |
| NLH2 | FAM-ATG CTA CAA AAC GGA CTT CGT AGC TCG GGG TTT AAA ATG AGC CAT-BHQ |
| NLH3 | TTT (FAM) GAA GGA TTG GCG AGA ATG GCT CAT TTT AAA GGC CTC GGA AAA TTC-BHQ |
| NLH4 | FAM-TCC GTT TTG GTT TCC GAA TTT TCC GAG GCC GGG TAC GCC TCG GGA-BHQ |
| LH1 | CTT CCT(BHQ) AAC CGC CCA CAA AGT TCA GCG GGG-FAM |
| LH2 | TTT (FAM)TGG GCG GTT AGG AAG CCC CGC TGA A(BHQ)CT TTG |
